# Molecular characterization of indigenous microbes and its potential as a biological control agent of Fusarium stem rot disease (*Fusarium verticillioides*) on maize

**DOI:** 10.1016/j.heliyon.2022.e11960

**Published:** 2022-11-30

**Authors:** Hishar Mirsam, Suriani, Muhammad Aqil, Muhammad Azrai, Roy Efendi, Ahmad Muliadi, Hasil Sembiring, Asti Irawanti Azis

**Affiliations:** a Research Center for Food Crops, National Research and Innovation Agency (BRIN), Cibinong Science Center—Botanical Garden, Jl. Raya Jakarta-Bogor No. Km. 46, Cibinong, Bogor Regency, 16911, Indonesia; b Ministry of Agriculture, Jl. Harsono RM. No. 3, Ragunan, Jakarta 12550, Indonesia; c Agriculture Faculty, Makassar Islamic University, Jl. Perintis Kemerdekaan Km 9. No. 29, Makassar, 90245, South Sulawesi, Indonesia

**Keywords:** Antagonist, Bacteria, Consortium microbes, Fungi, Molecular

## Abstract

*Fusarium* stem rot disease caused by *Fusarium verticillioides* has become one of the most serious issues confronting Indonesian farmers in recent years. An alternative option for suppressing this disease is to use indigenous microbes as an eco-friendly method to reduce synthetic fungicides. The objective of the research was to identify the molecular characteristics and effectiveness of an indigenous microbial consortium in controlling *Fusarium* stem rot disease. Identification of indigenous microbes is carried out based on molecular characters using universal primers, namely ITS-1/ITS-4 for fungi and 27F/1492R for bacteria. Nucleotide sequences were analyzed using Bioedit 7.2 version and MEGAX software. In vitro testing was carried out using the dual culture method for indigenous fungi and the disc diffusion method for indigenous bacteria. Meanwhile, in planta testing was conducted by evaluating a consortium of fungi and bacteria to control *F. verticillioides* in the field using a randomized block design with three replications, followed by a 5% DMRT test. The use of universal primer pairs ITS-1/ITS-4 and 27F/1492R succeeded in amplifying DNA bands of indigenous microbial isolates measuring ±600 bp and ±1465 bp, respectively. S6 and S9 bacterial isolates were identified as *Bacillus cereus*. JRP 7 and SEDF 6A isolates were identified as *Trichoderma asperellum* and JRP 10 isolate was identified as *Penicillium raperi*. All identification resulted in homology of >99%. The in vitro inhibitory reactions of isolates JRP 7, JRP 10, SEDF 6A, S6, and S9 against *F. verticillioides* were >60%. Disease severity of B6B9C10, B6B9C6, B6B9C7, B9C6C7, B9C7C10, and C6C7C10 treatments significantly proved their ability to control *F. verticillioides* in the field with a lower percentage of disease severity than positive controls, which are 23.33%, 18.89%, 23.33%., 21.85%, 14.07%, and 15.93%, respectively. The B9C7C10 consortium (S9 + JRP 7 + JRP 10 isolates) containing three species of indigenous microbes, i.e. *B. cereus, T. asperellum*, and *P. raperi* is the most effective at controlling *F. verticillioides* and may be developed for use as biopesticide products.

## 1 Introduction

Maize has strategic and economic importance as the main source of carbohydrates and protein after rice as well as a feed crop (Purwanto 2008; Khan et al., 2019). It is projected that by 2050, the demand for maize in emerging nations would climb by 70%–100%, and that by 2025, maize will emerge as the world's top-producing crop (Godfray et al., 2010). In Indonesia, maize is one of the most extensively produced food crops, with yields ranging from 5 to 7 tons per hectare (Ministry of Agriculture of the Republic of Indonesia 2018). This productivity is categorized as moderate, but there is significant potential for improvement given that, based on field study, greater yields of 10–14 tons per hectare are possible, depending on the variety and production strategy. Proper management/management of Plant Pest Organisms (PPO) is also required to achieve the yield potential of these varieties, in addition to the suitability of varieties with agro-ecosystem development.

Maize cultivation is inextricably linked to pest and disease issues, particularly plant pathogens. Maize crops are susceptible to a wide range of diseases, particularly those caused by pathogenic fungi. *Fusarium* stem rot disease, caused by *Fusarium verticillioides*, has recently attacked several Indonesian maize centers (Suriani et al., 2018; Mirsam et al., 2021; Pakki et al., 2021). This pathogen is reported to obstruct nutrients transported into plant tissues so that plant growth is inhibited and has a negative impact on filling of the cobs and rotting of the cobs (Zainuddin et al., 2017; Suriani et al., 2018). Damage caused by *Fusarium* stem rot disease ranged from 66.66–100.00% (Rahma et al., 2019). Besides infecting maize plants in the field, this pathogen is a pre-harvest fungus and contaminate maize seeds during drying and storage (Zhang et al., 2016; Wang et al., 2013).

Currently, *Fusarium* stem rot control still relies on synthetic chemical inputs of fungicides. The high input of synthetic chemicals, such as water pollution, causes eutrophication and poses a health risk to humans. Furthermore, this causes soil degradation and biodiversity loss (Roberts 2009). One alternative that can be used to reduce the use of synthetic chemicals is by utilizing beneficial microbes that can act as an eco-friendly alternative method to agricultural chemicals, where its application will increase agricultural sustainability (Lugtenberg et al., 2013; Suriani et al., 2021).

Indigenous microbes are groups of microorganisms from both beneficial fungi and bacteria that are aggressive and occupy the ecological niches of plants such as rhizosphere, rhizoplane, phylloplane, and endophytic microbes (Mirsam et al., 2015; Mirsam et al., 2016; Kandel et al., 2017). The indigenous microbial activity provides benefits for plant growth and development, either directly or indirectly. The direct effect is related to the ability of indigenous microbes to inhibit pathogenic activity by producing various compounds or metabolites such as antibiotics and siderophores as well as various antagonistic mechanisms (Ambrosini and Passaglia 2017; Mirsam et al., 2021). Meanwhile, the indirect effect is based on their ability to provide and mobilize or facilitate the absorption of various nutrients in the soil as well as to synthesize and change the concentration of various growth-promoting phytohormones (Simarmata et al., 2020).

In the past few decades, the role of indigenous microbes in sustainable agriculture and environmental management has received attention. The use of indigenous microbes in agroecosystems and solving major environmental problems has also shown remarkable results. In the agricultural sector, the use of indigenous microbes provides a conversion of low-input systems to high and more sustainable output systems. Several studies have shown that indigenous microbes have the potential to be used in controlling *F. verticillioides* as well as probiotic agents to induce plant growth in vitro and in planta (Saharan and Nehra 2011; Bhattacharyya and Jha 2012; Glick 2012; Kumar and Gopal 2015; Mirsam et al., 2021). Indigenous microbial isolates found in previous studies are still going through colony characterization, morphology, and physiology (Mirsam et al., 2021). Therefore, a fast and accurate characterization method is needed to determine the type of indigenous microbes. Identification through the molecular biology approach is believed to be faster and more accurate than the identification of morphological and physiological characters. One of the molecular techniques used is to amplify the ribosomal DNA in the internal transcribed spacers (ITS) for fungi and the 16S rRNA gene encoding for bacteria through the polymerase chain reaction (PCR) technique.

Molecular identification techniques based on total microbial DNA extraction provide a unique barcode for the determination and identification of different microbial isolates down to the species level (Landeweert et al., 2003). Molecular identification with these barcodes has shown to be a valuable tool for microbiologists investigating microbial taxonomy, molecular evolution, population genetics, and microbial-plant interactions (Möller et al., 1992). Microbial identification using molecular techniques was carried out by sequencing parts of target genes such as 16S rRNA for bacteria and the ITS region for PCR amplified fungi with universal primers for these microbial species (Monod et al., 2006; Henselt and Holden, 1996).

Conventional microorganism identification is performed by the culture method, followed by an examination of physiological characteristics and biochemical testing. Currently, a faster molecular-based identification method with high sensitivity and specificity has been developed. Molecular identification of indigenous microbes can be done with various molecular approaches. Identifying of spesific genes markers of a microbial taxon level has been widely developed. Analysis of the 16S ribosomal RNA (rRNA) gene and the internal transcribed spacer (ITS) ribosomal DNA (rDNA) region is the most frequently used identification method to identify bacteria and fungi (Alsohaili and Bani-Hasan 2018; Spina et al., 2021).

The 16S rRNA gene is a conserved gene sequence shared by all bacterial species. These gene sequences provide important information for the identification of all species. The 16S rRNA gene is very effective because it has high accuracy and fast identification (Fukuda et al., 2016). While the ITS rDNA sequence is an area that has relatively high sequence variations in the rDNA genes of each species, besides that ITS rDNA is also a region that evolves faster than other gene regions so that it will vary between species and is primely for identification at the species level (Rahayu 2015; White et al., 1990).

Currently, the use of biological agents in controlling plant pathogens is limited to a single application, making them less effective in the field. Considering the success rate of single fighter application of biological agents that have been used so far is still stagnant and tends to be ineffective, it is necessary to study the model of using multiple fighter-based biological agents or a consortium ofnumerous types of fungi and bacteria thatmay effectively and efficiently suppress plant pathogen attacks. The objective of this research was to examine the molecular characteristics of indigenous microbes and the effectiveness of the indigenous microbial consortium in controlling *Fusarium* stem rot disease.

## 2 Materials and methods

### 2.1 Purification and preparation of indigenous microbial isolates

The indigenous microbial isolates used in this study included 3 isolates of fungi collected from earlier studies, i.e SEDF 6A MRS, JRP 7 MRS, and JRP 10 MRS (Mirsam et al., 2021), as well as 2 isolates of bacteria (S6 and S9) from the collection of the Laboratory of Research Center for Food Crops, National Research and Innovation Agency. Furthermore, indigenous microbial isolates with single spored were purified and made stock by re-culturing on slant media of potato dextrose agar (PDA) for fungi and slant media of nutrient agar (NA) for bacteria using test tubes, then incubated at 28 °C for 7 days. After the fungus meets the sloping media, it is then stored in the refrigerator at 4 °C and used as stock for the next use.

### 2.2 Identification of indigenous microbe isolates by molecular characters

#### 2.2.1 Preparation of isolate indigenous microbe isolates suspension

The 7-days-old fungal isolates on PDA media were suspended using sterile distilled water. A total of 100 ml of sterile distilled water was added to the fungal isolates and then the conidia and mycelium were harvested using a spatula. Conidia and mycelium suspension of the indigenous fungi were put in collection bottles. Meanwhile, indigenous bacterial isolates were cultured on nutrient broth (NB) media. One loopful of indigenous bacterial isolate from the stock was put into an Erlenmeyer flask with 100 ml of NB media. The Erlenmeyer flask was then shaken on a rotary shaker at 180 rpm for 48 h at room temperature. The indigenous bacterial suspension was put in a collection bottle. Each suspension of fungi and bacteria was put into a 1.5 ml microtube and then centrifuged at 10,000 rpm for 5 min. The supernatant from the centrifuge was discarded while the precipitate/pellet was used for DNA extraction.

#### 2.2.2 Extraction of indigenous microbe DNA

DNA Extraction of indigenous microbe was carried out using the Zymo Research Quick-DNA Fungal/Bacterial Miniprep Kit. The working principle of this Zymo Research Quick-DNA Fungal/Bacterial Miniprep Kit is fungal and/or bacterial samples are added directly to a ZR BashingBead™ Lysis Tube (0.1 & 0.5 mm) and rapidly and efficiently lysed by bead beating without using organic denaturants or proteinases. The DNA is then isolated and purified using our Zymo-Spin™ Technology and is ideal for downstream molecular-based applications including PCR, array, etc.

#### 2.2.3 DNA amplification

DNA amplification was carried out by referring to the method carried out by Elfiati et al. (2019). The results of DNA extraction were followed by DNA amplification using the PCR method. The PCR reaction was carried out by mixing 12,5 μl of KAPA Taq ReadyMix PCR, 8,5 μl of ddH_2_O, 1 μl of primer forward, 1 μl of primer reverse, and 2 μl of DNA template. The primer used is a universal primer, i.e primer ITS-1 (5′-TCCGTAGGTGAACCTGCGG-3′) and ITS-4 (5′TCCTCCGCTTATTGATATGC-3′) for fungi and primer 27F (5′-AGAGTTTGATCCTGGCTCAG-3′) and 1492R (5′-TACGGYTACCTTGTTACGACTT-3′) for bacteria. The PCR cycle consists of an initiation or predenaturation step at 95 °C in 3 min which is followed by 35 cycles of the denaturation stage at 95 °C in 15 s, annealing stage at 55 °C in 30 s, extension stage at 72 °C in 1 min, and final extension stage in 3 min at 72 °C. Indigenous microbial DNA was electrophoresed at a voltage of 110 V for 50 min. The results of the electrophoresis were visualized with a UV transilluminator and photos were taken using the camera.

#### 2.2.4 Nucleotide sequence analysis

The amplification product is sent to FirstBase (Malaysia) for sequencing. Sequence results were analyzed using the basic local alignment search tool (BLASTN 2.13.0+) with an optimization program to obtain the sequence of DNA bases contained in the site of National Center for Biotechnology Information (NCBI). The nucleotide sequence results obtained were then analyzed using multiple alignment ClustalW on software Bioedit Sequence Alignment Editor 7.2 version. The kinship relationship between isolates was constructed using the software Molecular Evolutionary Genetic Analysis Software 10.0 version (MEGAX) with bootstrap 1000 times repetitions.

### 2.3 In vitro experiment: antagonis test of indigenous microbial isolates

#### 2.3.1 Dual culture antagonistic fungal activity test

The antagonistic activity test using the dual culture method was carried out by placing 5 mm diameter pieces of 7-days-old mycelium indigenous fungal isolates and *F. verticillioides* on PDA media in the same Petri dish. *Fusarium verticillioides* isolates were cultured 3 days earlier than the indigenous fungi. The position of each mold is arranged facing each other with a distance of 3 cm. Media that was inoculated only by *F. verticillioides* acted as control. Cultures were incubated at room temperature for 7 days accompanied by the observation of the radial growth of the colonies. Percentage growth inhibition (PGI) is determined based on Eq. (1):

(1) $$PGI=\frac{R1-R2}{R1}\times 100\%$$

where, PGI represented the inhibition percentage, R1 is radius *F. verticillioides* which grows against the antagonist microorganism, and R2 is the radius of the *F. verticillioides* colony that grows towards the antagonist microorganism (Seema and Devaki, 2012).

#### 2.3.2 Bacterial antagonistic test with disc diffusion method

A bacterial antagonist test using the disc diffusion method was carried out based on the method by Sua'rez-Estrella et al. (2006) that modified. Pure colonies of each indigenous bacterial isolate were taken using a needle and inoculated on NB medium in a test tube, and then incubated for 24 h at room temperature. After 24 h, the bacterial cell density was calculated using a hemocytometer. The bacterial cell density of each isolate was set at 106 cells mL-1 as the initial inoculant. A total of 0.1 mL of indigenous bacterial suspension was added to PDA media in a Petri dish and leveled by shaking to form a pattern like number 8. A 5 mm isolate of *F. verticillioides* was cultured on PDA media which had been mixed with indigenous bacteria and incubated at room temperature for 7 days. Indigenous bacterial isolates that have potential as antagonist agents are indicated by the presence of zones of inhibition on the growth of mycelium *F. verticillioides*. Each treatment was carried out with 3 repetitions. Observations were made on the measurement of the inhibition zone formed around the inoculum of *F. verticillioides* which indicated the presence of antimicrobial activity. The diameters of fungal growth in control and sample plates are measured.The bacterial antagonistic effect is estimated by the following formula (Eq. (2)):

(2) $$\text{PGI}=\frac{\text{Db}}{\text{Dc}}\times 100\%$$

where Dc is the diameter of growth in the control plate and Db is the diameter of growth in the plate containing tested bacterial antagonistic.

### 2.4 Compatibility test of indigenous microbe isolates

The compatibility of each indigenous microbe isolate in the consortium was tested using the antagonist test (Sarkar and Chourasia 2017). A compatibility test was carried out using PDA media. Indigenous fungi and bacteria were cultured together on PDA media, and then allowed to grow and develop until they filled the PDA media in Petri dish dishes. The observation indicator is that if one microbe forms a clear zone between microbes, it is indicated that the microbes are incompatible, and vice versa if the microbes grow and develop together without forming a clear zone, it is indicated that the microbes are compatible.

### 2.5 In planta experiment: consortium evaluation of indigenous microbe controlling *F. verticillioides* in field

#### 2.5.1 Multiplication of tested pathogen inoculum sources

Multiplication of *F. verticillioides* was carried out on sterile potato dextrose broth (PDB) media. Pure inoculum of *F. verticillioides* was propagated from a stock isolate of *F. verticillioides* which were first cultured on PDA media and incubated for 7 days at room temperature. Thereafter, the conidia were harvested from the 7-day-old culture of *F. verticillioides* and put into an Erlenmeyer flask containing 100 ml of PDB medium, then incubated for 4 days on a rotary shaker at 180 rpm at room temperature.

#### 2.5.2 Tested seed preparation

The seed soaking method was used to test the ability of the indigenous microbial consortium to suppress the attack intensity of *F. verticillioides* on maize crops in the field using Anoman variety maize seeds. Maize seeds were treated with hot water treatment at 55 °C for 20 min. The 7-days-old cultures of fungal isolates and the 48-hours-old bacterial isolates were suspended by adding sterile distilled water to the fungi and bacteria isolates, then scraped with a spatula and then used to soak the maize seeds. Maize seeds were soaked according to the indigenous microbial consortium treatment in Erlenmeyer containing a suspension of indigenous microbe isolates by shaking at 170 rpm for 24 h. The positive control treatment was carried out by soaking maize seeds that had been treated with hot water treatment using sterile distilled water, while the negative control treatment was soaked with a synthetic fungicide using active copper oxide 56%.

#### 2.5.3 Indigenous microbial consortium test on plants

##### 2.5.3.1 Planting material tested

The experimental land was set up in a full-tillage system by using a four-wheel tractor. Each treatment has a 3 × 5 m plot with 75 × 20 cm spacing and two seeds per hole. During planting and crop emergence, Furadan 3 G was applied at a rate of 8–10 kg/ha. First fertilization was at 7–10 days after planting (DAP) with 350 kg/ha NPK Phonska (15:15:15) and 150 kg/ha urea. After fertilizing, 25 plants per row were selected. The second fertilization was at 30 DAP with Urea at 250 kg/ha and NPK Phonska at 100 kg/ha.The third fertilization was at 45–50 DAP with Urea at 150 kg/ha, based on the leaf color chart. To ensure plant health, irrigation, weeding, pest and disease control were continuously monitored.

##### 2.5.3.2 Indigenous microbial consortium preparation and application

The fungi isolates, i.e SEDF 6A MRS, JRP 7 MRS, and JRP 10 MRS, were cultured and propagated on PDB media and then incubated at room temperature by shaking at 170 rpm for 120 h. Meanwhile, bacteria isolates, i.e S6 and S9, were cultured and propagated using NB media and incubated at room temperature by shaking at 170 rpm for 48 h. Fungal and bacterial isolates were consorted based on the treatment, using of each suspension of fungi (concentration 10^6^ conidia/mL) and bacteria (concentration 1 × 10^7−9^ colonies/ml) taken 100 ml and put into 700 ml PDB medium, then added with 10 g of molasses as microbial nutrition. The indigenous microbial consortium suspension was then incubated again at room temperature by shaking at 170 rpm for 48 h. Tested seeds were soaked in the microbial consortium based on each treatment at 160 rpm for 24 h. The soaked seeds were planted in the experimental field. Besides, the microbial consortium application was performed by spraying the suspension from the bottom to the top of the maize stem at 21, 28, 35 and 42 DAP. The indigenous microbe consortium treatments are (1) B6B9C6: Isolate S6 + S9 + SEDF 6A MRS + molasses; (2) B6B9C7: Isolate S6 + S9 + JRP 7 MRS + molasses; (3) B6B9C10: Isolate S6 + S9 + JRP 10 MRS + molasses; (4) B6C6C7: Isolate S6 + SEDF 6A MRS + JRP 7 MRS + molasses; (5) B6C6C10: Isolate S6 + SEDF 6A MRS + JRP 10 MRS + molasses; (6) B6C7C10: Isolate S6 + JRP 7 MRS + JRP 10 MRS + molasses; (7) B9C6C7: Isolate S9 + SEDF 6A MRS + JRP 7 MRS + molasses; (8) B9C6C10: Isolate S9 + SEDF 6A MRS + JRP 10 MRS + molasses; (9) B9C7C10: Isolate S9 + JRP 7 MRS + JRP 10 MRS + molasses; (10) C6C7C10: SEDF 6A MRS + JRP 7 MRS + JRP 10 MRS + molasses; (11) Control (-): Synthetic fungicide; (12) Control (+): Distilled water.

#### 2.5.4 Pathogen inoculation

The first inoculation of the pathogen *F. verticillioides* was performed when the plants were pre-silking, or 45–50 DAP, by injecting a 1 ml suspension of *F. verticillioides* (concentration 1 × 10^7−9^ colonies/ml) into the second stem segment. A week following the initial inoculation, the second inoculation was performed by injuring the second segment from the base of the stem with a cutter that had been dipped in suspension. *F. verticillioides*.

#### 2.5.5 Disease severity observation

Observation of the *Fusarium* stem rot disease severity was assessed on 90 DAP plants. Observations were made by taking 10 plant samples and scoring them based on the appearance of infection symptoms using the modified disease scale (Hooda et al., 2018). The standard rating scale of *Fusarium* stem rot disease on a standard of disease severity is as follows: Scale 0 [no infection]; Scale 1 [infection is confined to a very small spot in the pith at the site of inoculation (≤10%)]; scale 2 [infection spreads over a quarter of the inoculated internodes length in the pith and cortex tissue (>10–20%)]; scale 3 [infection spreads to half of the inoculated internodes length in the pith and cortex tissue (>20–30%)]; scale 4 [Infection spreads three-quarters of the inoculated internodes length in the pith and cortical tissue (>30–40%)]; scale 5 [infection covers the entire length of the inoculated segment, but does not cross the nodal plate. The skin is green and the symptoms are not visible from the outside, but the plant is showing signs of wilting (>40–50%)]; scale 6 [infection increases across the nodal plate and spreads to adjacent segments of the inoculated segment (>50–60%)]; scale 7 [infection spreads in two segments, the pith and cortex tissues degenerate, layer of the inoculated internodes is symptomatic and the plant wilts (>60–70%)]; scale 8 [infection spreads in three segments, the pith and cortex tissues degenerate, inoculated internodes are symptomatic and the plant wilts, the length and size of the ear are much reduced compared to healthy plants (>70–80%)]; and scale 9 [infection spreads over three segments; the pith, cortex tissue, and vascular bundles rot and become disorganized; olor skin changes, plants wilt a collapse (>80%)] (Figure S1). The disease scale is then transformed into the intensity percentage of disease formula as follows (Eq. (3)):

(3) $$I=\frac{\sum n\times v}{Z\times Ν}\times 100\%$$

where, I: disease severity index; n: scale frequency; v: score of rating scale; Z: maximal disease index/scale; N: total number of plants.

### 2.6 Re-isolation of the indigenous microbe pre-applied into maize plant

Soil from the rhizosphere and stem tissue of maize plants that were pre-applied with indigenous microbe were taken and weighed 10 g each. The soil and maize stem were crushed separately using a sterile mortar by adding 10 ml of sterile distilled water. Each Erlenmeyer flask added 10 mL of the suspension, 90 mL of distilled water and was homogenized for 30 min by a vortex. One millilitre of the homogenized suspension was diluted to 10^4^, 0.1 ml of the suspension was cultured on PDA medium for fungus and NA medium for bacteria. Multiple subcultures of microorganisms growing on each medium were repeated until a pure culture was produced and was suitable for identification.

### 2.7 Experiment design and data analysis

Antagonis test of indigenous microbial isolates was arranged using a completely randomized design. The treatments consisted of 5 indigenous microbe isolates, positive and negative control. Each treatment was replicated 3 times. Observational data were statistically analyzed with analysis of variance and followed by Least Significant Difference (LSD) at the 5% significance level (0.05). Meanwhile, the effectiveness of indigenous microbial consortium against *F. verticillioides* in the field using a randomized block design consisting of 10 consortium treatments, positive and negative control, then replicated three times. The data obtained were then statistically analyzed with analysis of variance and followed by Duncan's Multiple Range Test (DMRT) at the 5% significance level (0.05).

## 3 Results

### 3.1 Molecular characteristics of indigenous microbe isolates

**Figure 1:**
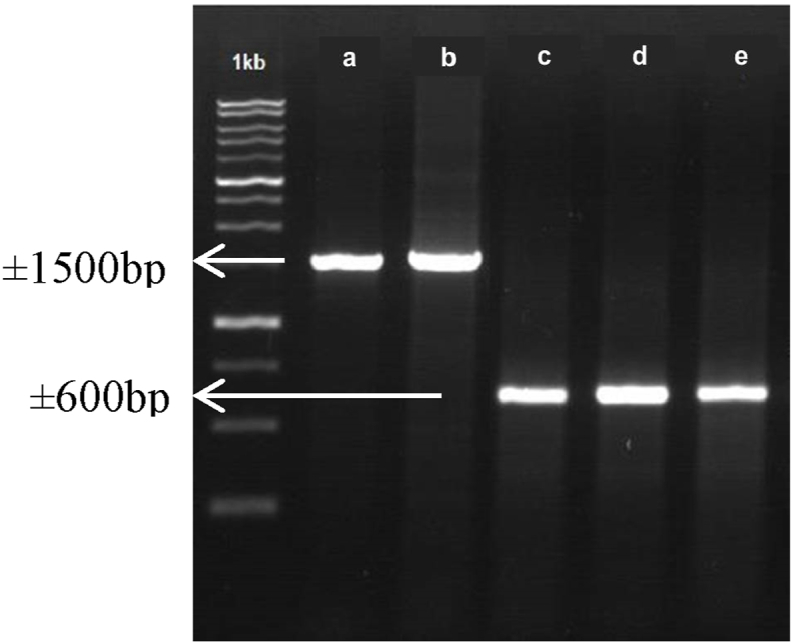
Agarose gel electrophoresis of polymerase chain reaction (PCR) amplification of indigenous microbe DNA with conditions 0.8% agarose gel, 0.1 μg of DNA ladder loaded per lane, and 1 μL of sample loaded per lane. 1 kb,Marker/DNA ladder; a, isolate S6; b, isolat S9; c, isolat JRP 7; d, JRP 10; e, isolate SEDF 6A.

The use of universal primer pairs ITS-1/ITS-4 and 27F/1492R successfully amplified DNA bands of indigenous microbe isolates measuring ±600 bp and ±1465 bp, respectively (Figure 1). The ITS-1/ITS-4 primer pair detected DNA in the internal region. transcribed spacer (ITS), which in this region does not encode functional proteins and is in the ribosomal RNA (rRNA) region. This region may be used as a genetic marker since sequence variation is rather substantial even within the same species, and all fungus possess ITS rRNA. While the universal primer pair 27F/1492R detected the presence of the 16S-rRNA encoding gene in S6 and S9 bacterial isolates, where this gene is known to be the skeleton of the ribosome which is generally used to identify bacterial groups.

The results of DNA sequence analysis of JRP 7, JRP 10, and SEDF 6A isolates based on ITS regional amplification revealed distinct identities for each of these fungal isolates with a similarity level of >99% (Table 1). The results of the analysis with the BLAST 2.13.0 + program are data showing the highest similarity (>90% with e-value 0.0) according to the determinations of Clavarie and Notre Dame (2003). The JRP 7 dan SEDF 6A isolates have similiarities to *Trichoderma asperellum* strain unilaGT from Lampung-Indonesia (99.7%), *T. asperellum*isolate WT13 from India (100%), *T. asperellum* strain MLT5J1 from Yogyakarta-Indonesia (100%), *T. asperellum* strain ACCC32915 from China (100%), and *T. asperellum* strain UC1 (MF774876) from Malaysia (99.7%). While the JRP 10 isolate have similarities to *Penicillium raperi* strain CMV006I1 from South Africa (100%), *P. raperi* strain DTO 249-D2 from Netherlands (100%), *P. raperi* strain IA32 from India (100%), and *P. raperi* clone SF_761 from China (100%). For the level of similarity between the sample isolates, JRP 7 and SEDF 6A, the similarity level was 100%, while the JRP 10 isolate had a similarity level of 72.70% to JRP 7 and SEDF 6A isolates.

**Table 1:** Identity matrix comparison of JRP 7, JRP 10, and SEDF 6A DNA sequences to GenBank isolates on NCBI.

Sequence code	JRP 7∗	JRP 10∗	SEDF 6A∗	A	B	C	D	E	F	G	H	I	J
JRP 7∗	ID												
JRP 10∗	0.727	ID											
SEDF 6A∗	1	0.727	ID										
A	0.997	0.729	0.997	ID									
B	1	0.727	1	0.997	ID								
C	1	0.727	1	0.997	1	ID							
D	1	0.727	1	0.997	1	1	ID						
E	0.997	0.729	0.997	1	0.997	0.997	0.997	ID					
F	0.727	1	0.727	0.729	0.727	0.727	0.727	0.729	ID				
G	0.727	1	0.727	0.729	0.727	0.727	0.727	0.729	1	ID			
H	0.727	1	0.727	0.729	0.727	0.727	0.727	0.729	1	1	ID		
I	0.727	1	0.727	0.729	0.727	0.727	0.727	0.729	1	1	1	ID	
J	0.391	0.365	0.391	0.391	0.391	0.391	0.391	0.391	0.365	0.365	0.365	0.365	ID

Description: A, *T. asperellum* strain unilaGT (LC650154) from Lampung-Indonesia, B, *T. asperellum* isolate WT13 (OL597956) from India; C, *T. asperellum* strain MLT5J1 (OK336354) from Yogyakarta-Indonesia; D, *T. asperellum* strain ACCC32915 (MF871569) from China; E, *T. asperellum* strain UC1 (MF774876) from Malaysia; F, *P. raperi* strain CMV006I1 (MK450713) from South Africa; G, *P. raperi* strain DTO 249-D2 (KC797647) from Netherlands; H, *P. raperi* strain IA32 (MZ267033) from India; I, *P. raperi* clone SF_761 (MT530037) from China; J, *B. bassiana* isolate ARSEF 1539 (DQ090720) from Germany. ∗) Research isolate.

The DNA nucleotide sequences of bacterial isolates based on the amplification of the gene encoding 16S revealed a similarity level of >99% between the S6 and S9 isolates and the genebank isolates in the NCBI (Table 2). The S6 isolate has similarities with *Bacillus cereus strain* WHX1 from China (99.9%), *B. cereus* strain YM16 from Malaysia (100%), *B. cereus* strain ABCFI from India (100%), *B. cereus* strain 156gite from France (99.8%), *B. cereus* strain NK1 from Japan (99.8%), *B. cereus* strain FORT 154 from Brazil (100%), *B. cereus*strain 44 from Argentina (100%), and *B. cereus* strain CZW-9 from China (100%). Meanwhile, S9 isolate had similiarities to *Bacillus cereus strain* WHX1 from China (99.8%), *B. cereus* strain YM16 from Malaysia (99.7%), *B. cereus* strain ABCFI from India (99.7%), *B. cereus* strain 156gite from France (99.9%), *B. cereus* strain NK1 from Japan (99.9%), *B. cereus* strain FORT 154 from Brazil (99.7%), *B. cereus* strain 44 from Argentina (99.7%), and *B. cereus* strain CZW-9 from China (99.7%). The level of similarity between S6 and S9 isolates was very high, 99.7%.

**Table 2:** Identity matrix comparison of S6 and S9 DNA sequences to GenBank isolates on NCBI.

Sequence code	S6∗	S9∗	A	B	C	D	E	F	G	H	I
S6∗	ID										
S9∗	0.997	ID									
A	0.999	0.998	ID								
B	1	0.997	0.999	ID							
C	1	0.997	0.999	1	ID						
D	0.998	0.999	0.999	0.998	0.998	ID					
E	0.998	0.999	0.999	0.998	0.998	1	ID				
F	1	0.997	0.999	1	1	0.998	0.998	ID			
G	1	0.997	0.999	1	1	0.998	0.998	1	ID		
H	1	0.997	0.999	1	1	0.998	0.998	1	1	ID	
I	0.879	0.88	0.88	0.879	0.879	0.881	0.881	0.879	0.879	0.879	ID

Description: A, *B. cereus* strain WHX1 (MN216227) from China; B, *B. cereus* strain YM16 (KP686225) from Malaysia; C, *B. cereus* strain ABCFI (MN121339) from India; D, *B. cereus* strain 156gite (MT383675) from France; E, *B. cereus* strain NK1 (AB295052) from Japan; F, *B. cereus* strain FORT 154 (MG561368) from Brazil; G, *B. cereus* strain 44 (MT783984) from Argentina; H, *B. cereus* strain CZW-9 (MG866080) from China; and I, *B. subtilis* strain IJ114 (MT071635) from Iran. ∗) Research isolate.

**Figure 2:**
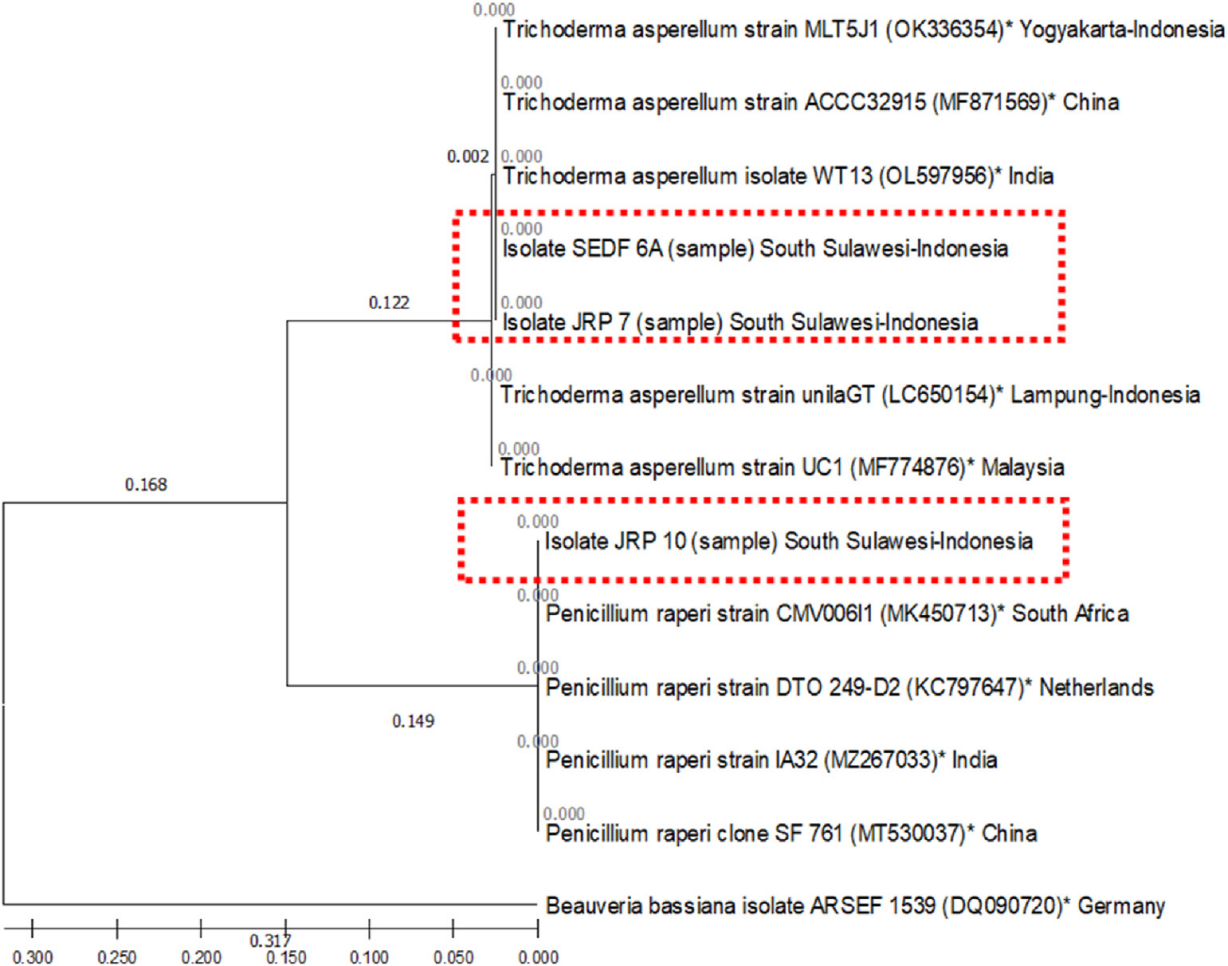
Phylogenetic tree of JRP 7 MRS, JRP 10 MRS, and SEDF 6A fungal isolates based on Neighbor-Joining analysis with genetic distance matrix calculations implemented in the Bioedit 7.2 and MEGAX programs. The scale below the figure is a scale of genetic distance coefficient values that describes the average number of nucleotide changes among isolates. ∗) Accession number.

Phylogenetic analysis showed that JRP 7 and SEDF 6A isolates were in the same group as *T. asperellum* isolate WT13 from India, strain ACCC32915 from China, and strain MLT5J1 from Yogyakarta-Indonesia with a genetic distance coefficient of 0.000. In addition, JRP 7 and SEDF 6A isolates also showed close kinship. which is close to *T. asperellum* strain unilaGT from Lampung-Indonesia and strain UC1 from Malaysia with a genetic distance coefficient of 0.002. While JRP 10 isolates had a very close relationship to *P. raperi* strain CMV006I1 from South Africa, strain DTO 249-D2 from Netherlands, strain IA32 from India, and clone SF_761 from China with a genetic distance coefficient of 0.000. The JRP 7 dan SEDF 6A isolates had a fairly distant kinship to the JRP 10 isolate which is indicated by the coefficient of genetic distance that is far from 0.122 to 0.149 (Figure 2).

**Figure 3:**
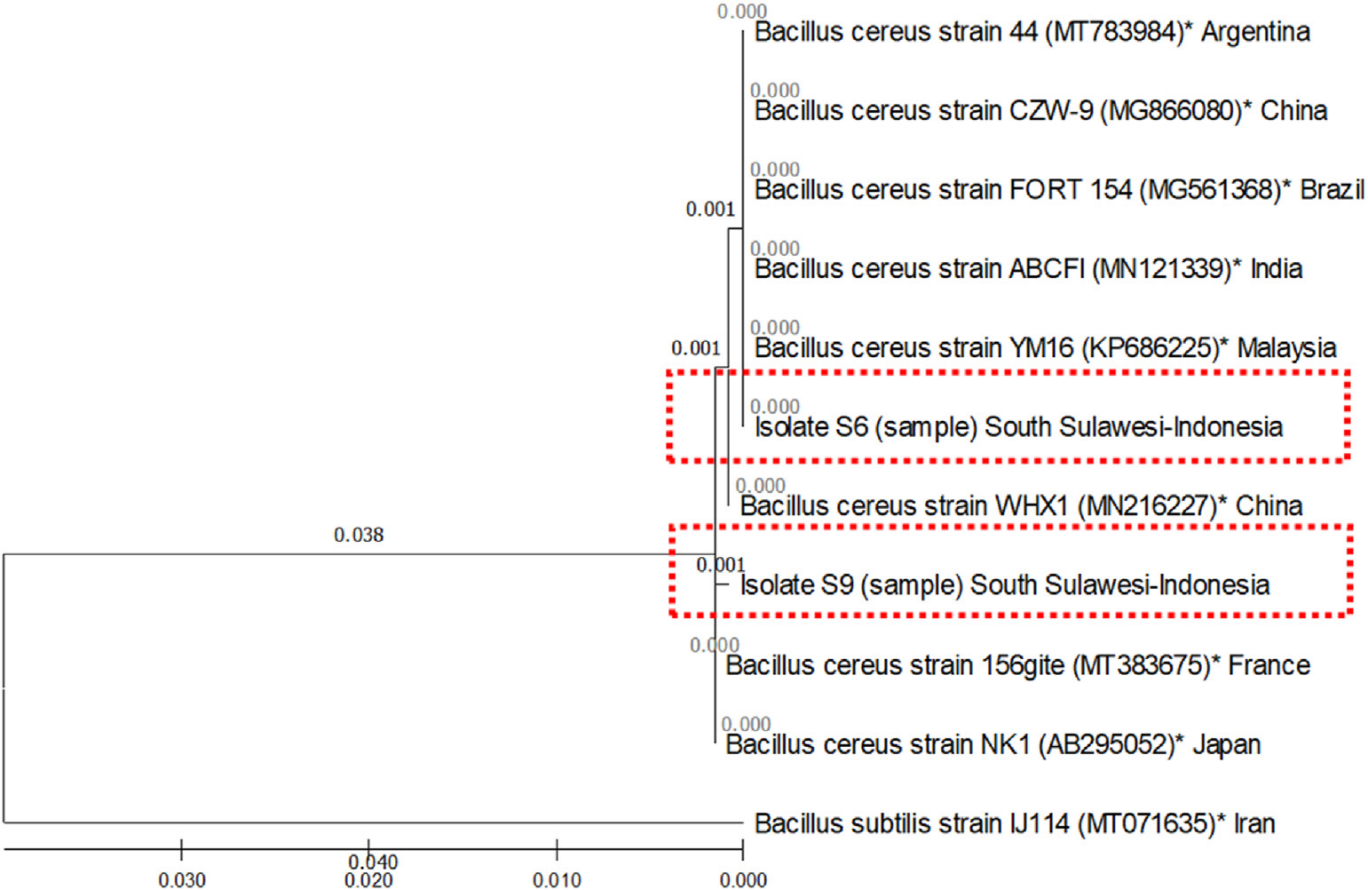
Phylogenetic tree of S6 and S9 bacterial isolates based on Neighbor-Joining analysis with genetic distance matrix calculations implemented in the Bioedit 7.2 and MEGAX programs. The scale below the figure is a scale of genetic distance coefficient values that describes the average number of nucleotide changes among isolates. ∗) Accession number.

The S6 isolate was closely related to *B. cereus* strain YM16 from Malaysia, ABCFI strain from India, FORT strain from Brazil, ZCW-9 strain from China, and strain 44 from Argentina with a genetic distance coefficient of 0.000. Meanwhile, S9 isolate had a very close kinship with *B. cereus* strain 156gite from France and strain NK1 from Japan with a genetic distance coefficient of 0.000. In addition, S6 and S9 isolates also showed a close kinship level with a genetic distance coefficient of 0.001 (Figure 3).

### 3.2 Inhibition of indigenous microbe isolates against *F. verticillioides* in-vitro test

**Figure 4:**
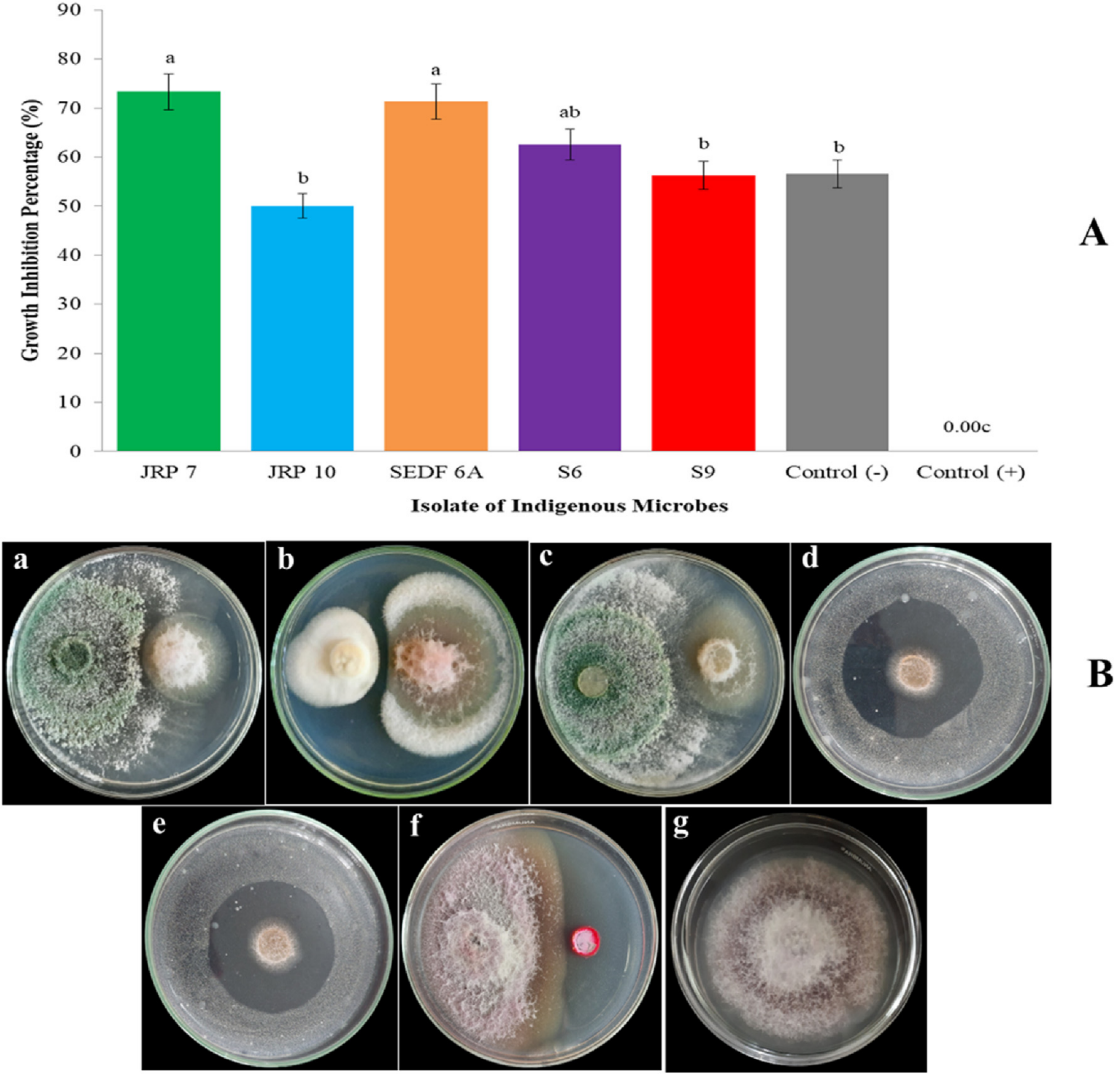
Inhibition of indigenous microbe isolates against *F. verticillioides* on PDA media. A) Graph of growth inhibition percentage. The same letter in the graph are not significantly different according to the LSD test at the 5% level. B) In-vitro interaction between indigenous microbes and *F. verticillioides*. a, JRP 7 fungal isolate; b, JRP 9 fungus isolate; c, SEDF 6A fungus isolate; d, S6 bacterial isolate; e, S9 bacterial isolate; f, *F. verticillioides* against synthetic fungicide as a negative control; g, *F. verticillioides* as a positive control plate. a, b, c, f, dual culture method; d, e, disc diffusion method.

The findings indicated that the five indigenous microbial isolates inhibited the growth of *F. verticillioides* in vitro on PDA medium with a 50% inhibition rate. Inhibition of JRP 7, JRP 10, SEDF 6A, S6, and S9 isolates against *F. verticillioides* were 73.33%, 50%, 71.43%, 62.50%, and 56.25%, respectively (Figure 4A). It was shown that inhibition was much greater than the positive control level of 0.00%. The JRP 7 and SEDF 6A fungal isolates inhibited the growth of *F. verticillioides* with a parasitism mechanism, in which there was competition for growth between the antagonists and the pathogen tested for nutrients and limited space, which caused the *F. verticilliodes* pathogen cannot be able to grow properly. Meanwhile, the JRP 10 fungus isolate and S6 and S9 bacterial isolates inhibited the growth and development of *F. verticillioides* through an antibiosis mechanism by showing the formation of an inhibition zone between the test isolate and the pathogen *F. verticillioides* (Figure 4B).

### 3.3 Compatibility of indigenous microbe isolates

The compatibility test results of 5 selected microbial isolates showed that all isolates were a mutually compatible relationship, except for SEDF6A and JRP7 isolates which showed incompatible relationships on PDA media (Table 3, Figure S2). The incompatible relationship between isolates SEDF6A and JRP7 was indicated by an invasion/replacement reaction, where one fungal colony grew towards another fungus and the hyphae coiled each other (Figure S2c). There were 10 consortiums of fungi and bacteria that became candidates for mixed culture (the consortium) based on the results of the suitability test (compatibility) and hemolysis, namely the consortium code B6B9C10, B6B9C6, B6B9C7, B6C6C10, B6C6C7, B6C7C10, B9C6C10, B9C6C7, B9C7C10, and C6C7C10. The 10 fungi and bacteria consortiums were compatible with each other and non-pathogenic from pathogenicity andhemolysis testing.

**Table 3:** Results of indigenous microbe compatibility test.

Field Isolate Code	S6	S9	SEDF 6A	JRP 7	JRP 10
S6	+	+	+	+	+
S9	+	+	+	+	+
SEDF 6A	+	+	+	−	+
JRP 7	+	+	−	+	+
JRP 10	+	+	+	+	+

Description: (+) compatible; (−) incompatible.

### 3.4 Effect of indigenous microbes consortium on Fusarium stem rot disease intensity in the field

**Figure 5:**
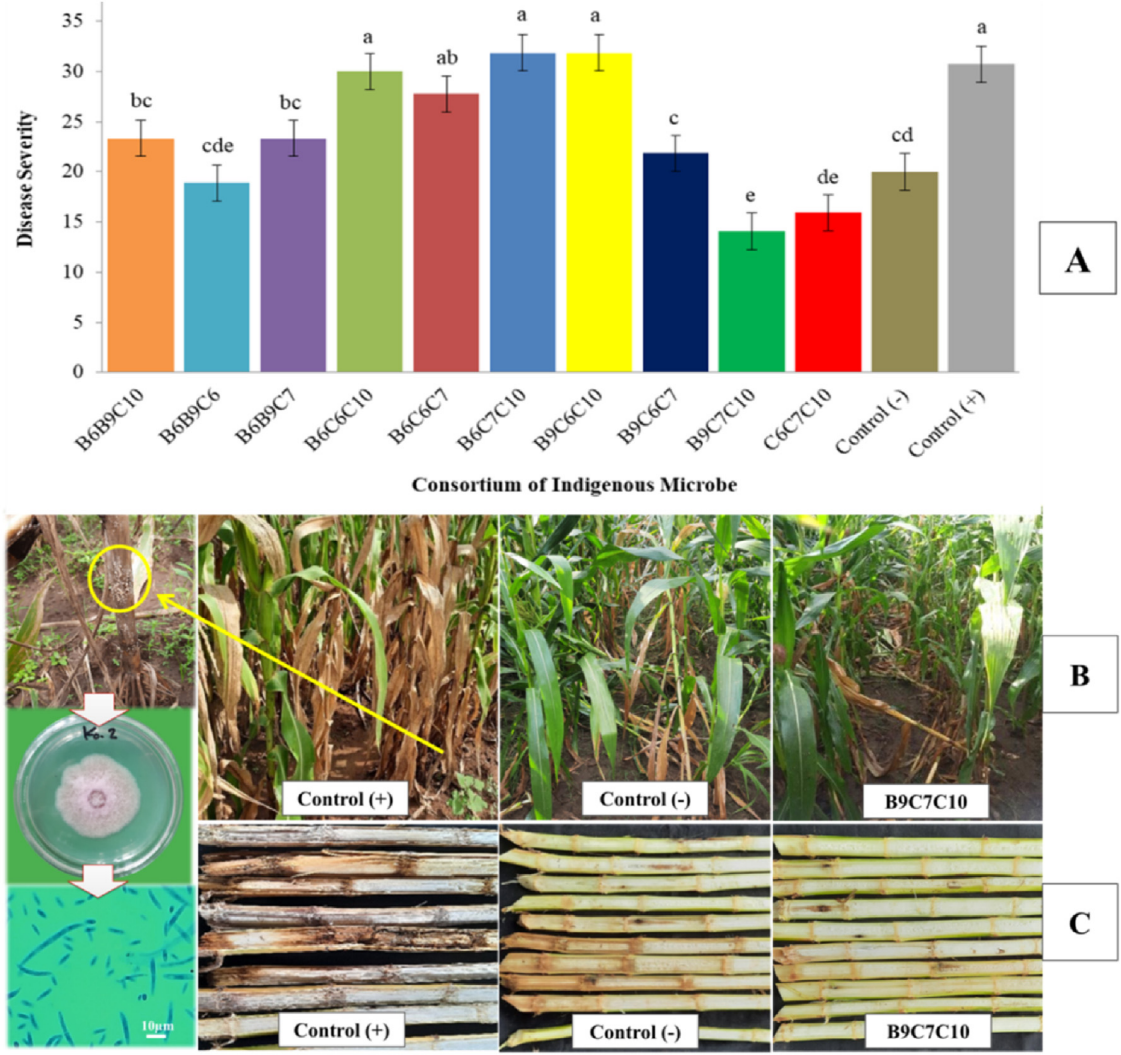
The effect of the indigenous microbes consortium on the *Fusarium* stem rot severity. A) Graph of *Fusarium* stem rot disease severity percentage. The same letter in the graph are not significantly different according to the DMRT test at the 5% level. B) symptoms of *Fusarium* stem rot disease in maize crops. C) Symptoms of *Fusarium* stem rot disease in the pith and cortex tissue of maize stems.

The average severity of *Fusarium* stem rot disease in maize revealed that several microbial consortium treatments (between bacteria and fungi) had a similar potential as negative controls using chemical-synthetic fungicides (Nordox® 56WP) to suppress the development of *Fusarium* stem rot disease in the observation field. Statistical analysis showed that several microbe consortium treatments were significantly different from the positive control (distilled water) to suppress *Fusarium* stem rot disease at 5% DMRT test. Disease severity of B6B9C10, B6B9C6, B6B9C7, B9C6C7, B9C7C10, dan C6C7C10 treatments were 23.33%, 18.89%, 23.33%, 21.85%, 14.07%, dan 15.93%, respectively. It was showed significantly that the disease severity was lower than the positive control, 30.74% (Figure 5A).

### 3.5 Confirmation of the presence of indigenous microbe isolate on maize plant

Three indigenous microbes that were successfully re-isolated from the tested plants were then identified visually under a microscope. The microbes were identified as their original microbial isolates, i.e. *T. asperellum*, *P. raperi*, and *B. cereus* (Figure S3a, S3d). Isolate JRP 7 had greenishwhite colony color that turns dark green when approaching old age, hyaline hyphae with a septum, and pyramidal conidiophore with the phialide branches, the phialide size, conidia globose-shaped and green, and growth rate of 34.43 mm/day (Figure S3). The characteristics of isolate JRP 7 were similar to *T. asperellum* characterized by Oszako et al. (2020), i.e. *T. asperellum* had conidiophores to which branched phialide, 2–3 in number, measuring 6.4 × 1.4 μm. Conidia were colored greenish, ellipsoid, 3.1 × 2.6 μm, and chlamydospores unicellular, terminal, and roughly spherical measuring 20.5 × 21.2 μm. The colony of *Penicillium* sp. has a cotton-like surface texture with radial grooves and yellowish-white color, and reached maximum growth on day 9th on PDA media. The morphological characteristic of *Penicillium* sp. was a single-celled chain of conidia produced from specialized conidia cells called phyalids (Figure S3b, S3e). These characteristics are comparable to those reported by Visagie et al. (2014), who described the colonies as having a yellowish-white hue with a thick, velvety surface. Septate hyphae, branched mycelium, typically colorless, septate conidiophores, branched or unbranched hyphae, broom-like of hyphae heads that bearing conidia, sterigmata appearing in clusters, chain-shaped of conidia which because it appears one by one from the sterigmata. Conidium is green, then turns bluish or brownish. Meanwhile, the growth of *B. cereus* on NA culture media showed that the morphology of *B. cereus* colonies was milky white and irregular shaped with a convex and slightly shiny surface. *B. cereus* has short rod-shaped cells to single rods with a single arrangement (Figure S3c, S3f). Safitri et al. (2020) described the characteristics of *B. cereus* bacteria, i.e white, circular shape, short bacilli with all edges, and convex elevation.

## 4 Discussions

Amplification of ITS rDNA sequences of indigenous fungi using ITS-1/ITS-4 primer pair followed by nucleotide sequencing succeeded in identifying isolates of JRP 7, JRP 10, and SEDF 6A. The JRP 7 and SEDF 6A isolates were identified as *T. asperellum* with a homology level of >99% and a genetic distance coefficient of 0.000–0.0001. Meanwhile, JRP 10 isolate was identified as *P. raperi* with 100% homology and a very close genetic distance coefficient of 0.000. Fungal DNA amplification by PCR technique using ITS-1/ITS-4 primer pair amplifies the ITS rDNA region. The ITS region is the genomic coding region for the rDNA components. In addition, this ITS region also has a high probability of identifying the type of fungus because it has the clearest barcode differences between species or there are inter- and intra-specific DNA variations (Schoch et al., 2012; Poczai et al., 2014; Zhang et al., 2016; Buehler et al., 2017). The ITS rDNA region is widely used in fungal phylogenetics, classification, and identification due to its universal nature, conservative sequence structure, and abundance (Legiastuti and Aminingsih 2012).

The 16 rRNA gene from isolates S6 and S9 was successfully amplified using primer pairs 27F and 1492R as indicated by the appearance of ±1600 bp carved DNA bands on the agarose gel. DNA amplification of S6 and S9 bacterial isolates was followed by sequencing analysis which showed the two isolates as *B. cereus* bacteria with homology values > 99% and genetic distance coefficient 0.000–0.001. The analysis of the 16S rRNA gene used in this study is similar to that of Shah et al. (2022) and as an indicator for the universal identification of microorganisms with a size of approximately 1500 bp. Thus, the identification of a bacterium can be done by amplifying the 16S rRNA region of the bacterial genome.

Molecular characteristics were further confirmed to ensure that these indigenous microbe species have the potential as biological agents. Characterization of potential antagonists of JRP 7, JRP 10, SEDF 6A, S6, and S9 isolates is the first step in evaluating the capacity of these isolates as biological agents. The test results revealed that 50% of the five isolates inhibited the growth of *F. verticillioides* in vitro test on PDA medium, classifying them as antagonistic agents. In line with the theory that the threshold of antagonistic fungi can inhibit pathogenic fungi, according to Otter et al. (2004), i.e. if the percentage of inhibition reaches 30% of the surface of the Petri dish, then the antagonist fungus only has a minimal inhibitory effect on the growth of pathogenic fungi. However, if the inhibition covers more than 60% of the Petri dish's surface, it should be possible for the antagonist fungus to inhibit the development of pathogenic fungi.

Indigenous microbe isolates inhibited the growth of *F. verticillioides* with various mechanisms. The isolates of JRP 7A and SEDF 6A which were identified as *T. asperellum* were able to inhibit the pathogen tested by a competition mechanism. *Trichoderma* spp. is a type of biological agent capable of controlling plant pathogens due to the production of biologically active compounds such as alkaloids, paxillins, lolitrems, and steroid tetranone (Sudhanta and Abadi 2011; Carreras-Villasen et al., 2012). In addition, *Trichoderma* spp. as an antagonist agent has an inhibitory mechanism in the form of competition that occurs between two types of fungi that are grown side by side. This rivalry arises because all fungus require a place to grow and the nutrient-rich medium used to grow plants (Ara et al., 2012). A number of studies have reported that *T. asperellum* acts as a biological agent for soil-borne pathogens through various antagonistic mechanisms such as parasitism, competition, and antibiosis and reduces pathogen infection by about 60% in vitro test (Begoude et al., 2007; Sant et al., 2010; Mbargaa et al., 2012; Zhang et al., 2021).

The JRP 10 isolate which was identified as *P. raperi* showed its ability to inhibit *F. verticillioides* by an antibiosis mechanism. This type of fungus has been reported as a biological agent in several plant pathogens. A number of studies had also shown that this *Penicillium* species has an impressive range of biotechnological potentials, such as being able to produce enzymes, biocontrol agents, plant growth promoting agents, bioremediation, biodegradation, biotransformation, biosynthesis and cycling nutrition (Strobel et al., 2004; Carvalho et al., 2012; Zhang et al., 2014; Suman et al., 2016).

The S6 and S9 bacterial isolates inhibited the pathogen *F. verticillioides* in vitro with an antibiosis mechanism. The S6 and S9 isolates, which were identified as *B. cereus*, had antibiosis abilities because they produced compounds that interfered with the morphological and physiological growth of pathogenic fungi. There are several ways to inhibit the attack of pathogenic fungi by bacteria (Purwantisari et al., 2005; Tasnim et al., 2011; Malinda et al., 2015). First of all, bacteria produce bioactive compounds that can degrade the structural components of fungi. These compounds degrade fungal cell walls. Second, bioactive compounds affect the permeability of fungal cell membranes, interfering with the transport of metabolically important substances. This causes the metabolism of the fungus to be disturbed. Third, compounds produced by bacteria can work as inhibitors of an enzyme produced by fungi. If these enzymes play an important role in the fungi's metabolism, the enzymatic activity of the cells will be messed up. As a result, it will inhibit the growth of fungi. Fourthly, bacteria-produced compounds can inhibit protein synthesis in fungi. When protein synthesis is interrupted, the fungus doesn't make enough of some proteins, which slows its growth.

The use of a single biological agent is considered to be less effective, so in this study, a model for the use of multiple fighter-based biological agents was carried out by consortium of several types of fungi and bacteria that can suppress the development of *Fusarium* stem rot disease. Compatibility testing of indigenous fungi and bacteria isolates in the laboratory resulted in 10 consortium combinations that were mutually compatible and non-pathogenic, i.e. B6B9C10, B6B9C6, B6B9C7, B6C6C10, B6C6C7, B6C7C10, B9C6C10, B9C6C7, B9C7C10, and C6C7C10. Resti et al. (2018) explained that to create a microbial consortium, the microbial cultures used must be compatible with each other. In addition, the microbes that will be used as biological agents are not pathogenic in humans and animals (secure) when applied in the field.

Statistic analysis showed that consortium treatment of B9C7C10 (microbes combination of *T. asperellum*, *P. raperi*, *B. cereus*) suppressed the density disease of *Fusarium* stem rot with the lowest attack percentage of 14.07% by 5% DMRT test. This value indicated a significantly lower disease severity compared to the positive control (30.74%). In the positive control treatment, *Fusarium* stem rot was seen much more than in the microbial consortium treatment with symptom characteristics, that the lower stem was yellowish and in severe attacks it turned fawn-colored. In addition, symptomatic plants also showed gray and drooping leaves (Figure 5B, 5C). Similar symptoms were also reported by Soenartiningsih et al. (2016) that the initial symptom of disease infected with *Fusarium* fungus on maize plants is sudden wilting of the leaves. Within one to two days, the leaves get gray and wilt. If the infection is heavy, the bottom stem's color will shift to fawn. The stem of the lowest internode has a rotting pith and detaches from the outer bark of the stem, then the stem becomes mushy.

The direct evaluation results in the field showed that the B9C7C10 consortium had the best suppression of disease severity because the *T. asperellum, P. raperi, B. cereus* contained in this consortium were able to colonize the roots well, so that the plant roots performed their functions optimally and indirectly induce plant resistance. Notodarmojo (2005) explained that some of the advantages of using a microbial consortium are (1) sequential degradation can be carried out, (2) the required enzyme or substance can be produced, (3) the overall substrate degradation rate can be increased, (4) oxidation is facilitated, because it can find the easiest thermodynamic pathway.

The colonization of rhizosphere microorganisms in plant roots plays an important role in plant nutrition, competition, and disease resistance (Kloepper and Beauchamp 1992). This symbiotic relationship can effectively activate plant resistance responses locally and systemically so that plants respond more effectively to potential biotic and abiotic stresses (Jung et al., 2012; La Spada et al., 2020). In addition, the relatively poor effect of biological agents in some of the treatments observed in this study is usually associated with the failure of colonization by antagonistic fungi and bacteria applied as stated by Benizri et al. (2001). In this study, it was shown that the combination of *T. asperellum, P. raperi,* and *B. cereus* consortium succeeded in colonizing the maize rhizosphere area well, which was indicated by the lowest intensity of *Fusarium* stem rot disease.

## 5 Conclusions

Bacterial isolates of S6 and S9 were identified *as B. cereus* with >99% homology. The JRP 7 and SEDF 6A isolates were identified as *T. asperellum* and JRP 10 isolate was identified as *P. raperi* with >99% homology. Isolates of JRP 7, JRP 10, SEDF 6A, S6, and S9 showed inhibitory reactions to *F. verticillioides* in vitro with >60% of inhibition percentage. The B9C7C10 consortium (S9 + JRP 7 + JRP 10 isolates) with indigenous microbes of *B. cereus, T. asperellum,* and *P. raperi* was most effective against *F. verticillioides* that the lowest percentage of disease severity is 14.07%. Considering the field success of multiple fighter-based biological agents, this microbial consortium can be further developed as a biopesticide formulation.

## Declarations

### Author contribution statement

All authors listed have significantly contributed to the development and the writing of this article.

### Funding statement

Muhammad Aqil and research members were supported by The National Research and Innovation Agency of the Republic of Indonesia through the funding research support on the National Research Priority Grant Programme (PRN 2021).

### Data availability statement

Data included in article/supp. material/referenced in article.

### Declaration of interest’s statement

The authors declare no conflict of interest.

### Additional information

Supplementary content related to this article has been published online at [URL].
