# Supplementary material for: Molecular characterization of indigenous microbes and its potential as a biological control agent of Fusarium stem rot disease (Fusarium verticillioides) on maize
Source: Heliyon. 2022 Nov 30;8(12):e11960. doi: 10.1016/j.heliyon.2022.e11960 (PMC9720014; doi:10.1016/j.heliyon.2022.e11960)
Supplement: Supplementary File [file mmc1.docx]

**Supplementary File**


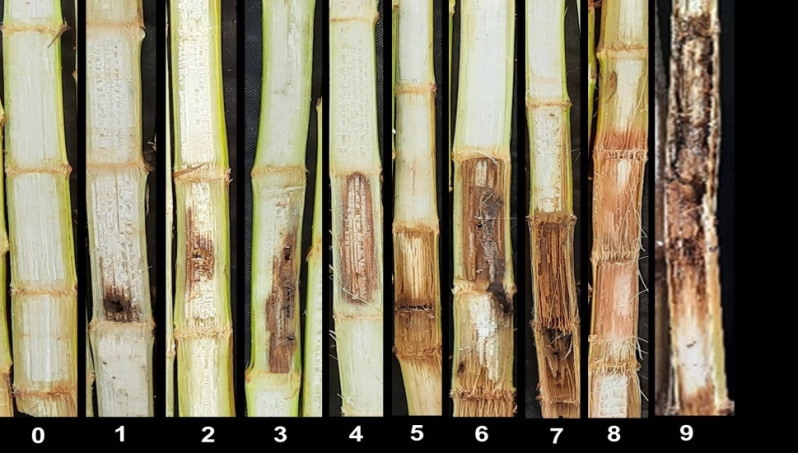


**Figure S1.** Measurement of Fusarium stem rot disease on standard rating scale and determination of disease reaction


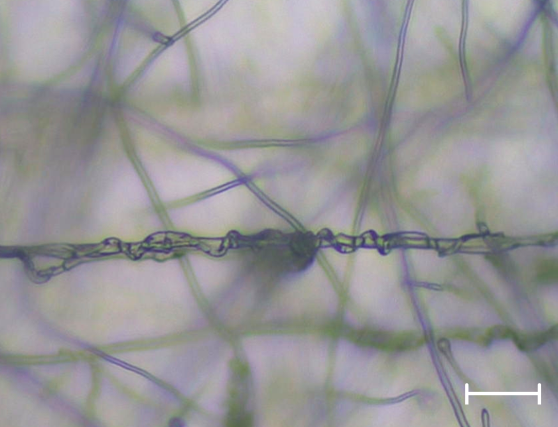


**c**


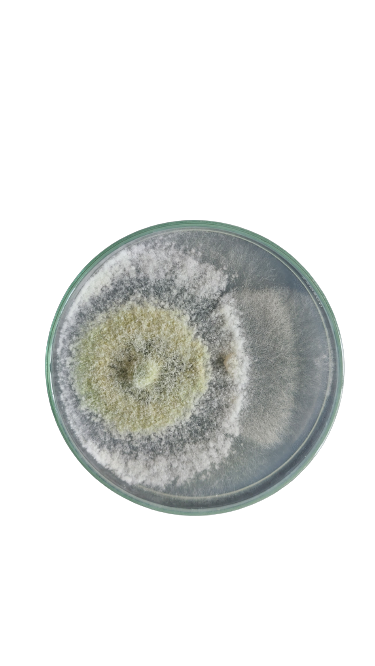


**b**


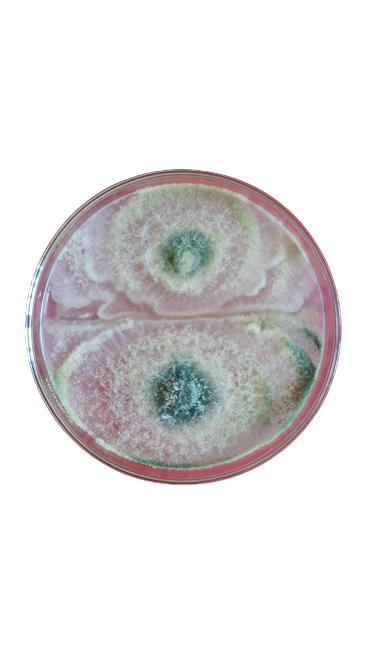


**a**

**Figure S2.** Synergistic interaction between isolates. a, compatible; b, incompatible; c, hyphae coiled each other (bar scale= 100 μm).


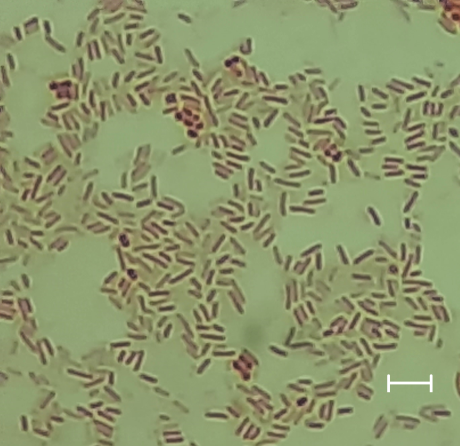


**f**


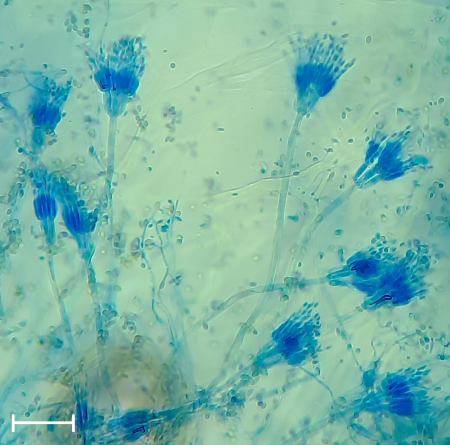


**e**


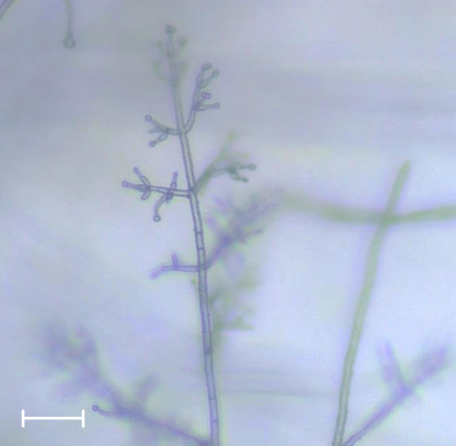


**d**


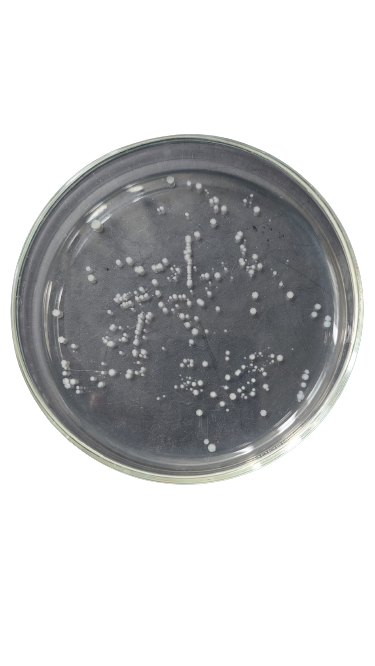


**c**


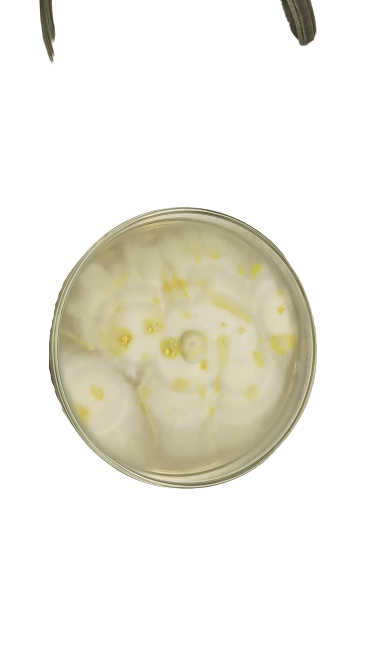


**b**


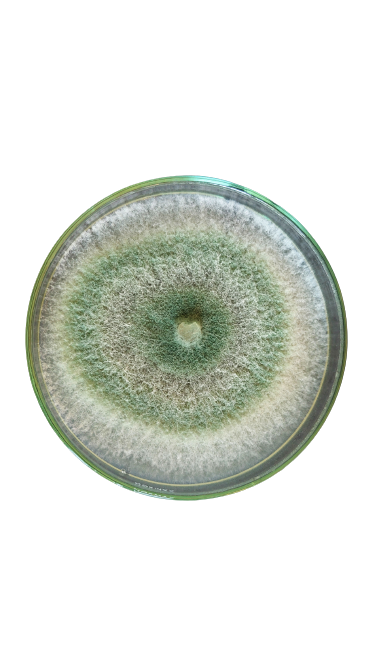


**a**

**Figure S3.** Morphology of fungal indigenous microbe isolates from re-isolation of rhizosphere and maize stem tissue. *Trichoderma asperellum* isolate: (a) 4 days old culture plate on PDA, (d) microscopic characters (bar scale = 50 μm). *Penicillium raperi* isolate: (b) 12 days old culture plate on PDA, (e) microscopic characters (bar scale = 50 μm). *Bacillus cereus* isolate: (a) 24 hours old culture plate on NA, (d) microscopic characters (bar scale = 5 μm).
